# Supplementary material for: Ursolic Acid-Based Nutraceutical Mitigates Muscle Atrophy and Improves Exercise Performance in Mouse Model of Peripheral Neuropathy
Source: Int J Mol Sci. 2025 Jun 5;26(11):5418. doi: 10.3390/ijms26115418 (PMC12155626; doi:10.3390/ijms26115418)
Supplement: Supplementary file 1 [file ijms-26-05418-s001.zip › ijms-3579588-supplementary.pdf]

Supplementary materials

Table S1. Oligonucleotides used for real-time PCR

Oligonucleotides used for real-time PCR

Gene	**Forward primer (5' → 3')**	**Reverse primer (5' → 3')**
CypA	CGCCACTGTCGCTTTTCG	AACTTTGTCTGCAAACAGCTC
Atrogin-1	CCTGCATGTGCTCAGTGAGGA	CTTCTTGGGTAACATCGTACAAGC
Murf-1	ACCTGCTGGTGGAAAACATC	CTTCGTGTTCCTTGCACATC
IL-6	TGAACAACGATGATGCACTTG	TCTCTCTGAAGGACTCTGGC
IL-8	TGGGTGAAGGCTACTGTTGG	AGCTGACTTCACTGGAGTCC
TNF-a	CAGAAAGCATGATCCGCGAC	GGTCTGGGCCATAGAACTGA
CXCL-16	AGTGGGTCCGTGAACTAGTG	GGAAGAGTGGAGTGCTGAGT
CXCR-4	CTGGCCTTCATCAGCCTGGA	ATGTCCCCCTGCCTGACGTC
SOD-1	ATGTTGGAGACCTGGGCAAT	CCACCTTTGCCCAAGTCATC
SOD-2	ATCAGGACCCATTGCAAGGA	AGGTTTCACTTCTTGCAAGCT
